# 3D Lamellar-Structured Graphene Aerogels for Thermal Interface Composites with High Through-Plane Thermal Conductivity and Fracture Toughness

**DOI:** 10.1007/s40820-020-00548-5

**Published:** 2020-11-11

**Authors:** Pengfei Liu, Xiaofeng Li, Peng Min, Xiyuan Chang, Chao Shu, Yun Ding, Zhong-Zhen Yu

**Affiliations:** 1 Beijing Key Laboratory of Advanced Functional Polymer Composites, Beijing University of Chemical Technology, Beijing, 100029 People’s Republic of China; 2 State Key Laboratory of Organic-Inorganic Composites, College of Materials Science and Engineering, Beijing University of Chemical Technology, Beijing, 100029 People’s Republic of China; 3 Beijing Advanced Innovation Center for Soft Matter Science and Engineering, Beijing University of Chemical Technology, Beijing, 100029 People’s Republic of China

**Keywords:** Anisotropic aerogels, Graphene, Thermal conductivity, Epoxy composites, Fracture toughness

## Abstract

**Highlights:**

- Lamellar-structured graphene aerogels with vertically aligned and closely stacked high-quality graphene lamellae are fabricated.
- The superior thermally conductive capacity of the aerogel endows epoxy with a high through-plane thermal conductivity of 20.0 W m^−1^ K^−1^ at 2.30 vol% of graphene content.
- The nacre-like structure endows the epoxy composite with enhanced fracture toughness.

**Abstract:**

Although thermally conductive graphene sheets are efficient in enhancing in-plane thermal conductivities of polymers, the resulting nanocomposites usually exhibit low through-plane thermal conductivities, limiting their application as thermal interface materials. Herein, lamellar-structured polyamic acid salt/graphene oxide (PAAS/GO) hybrid aerogels are constructed by bidirectional freezing of PAAS/GO suspension followed by lyophilization. Subsequently, PAAS monomers are polymerized to polyimide (PI), while GO is converted to thermally reduced graphene oxide (RGO) during thermal annealing at 300 °C. Final graphitization at 2800 °C converts PI to graphitized carbon with the inductive effect of RGO, and simultaneously, RGO is thermally reduced and healed to high-quality graphene. Consequently, lamellar-structured graphene aerogels with superior through-plane thermal conduction capacity are fabricated for the first time, and its superior through-plane thermal conduction capacity results from its vertically aligned and closely stacked high-quality graphene lamellae. After vacuum-assisted impregnation with epoxy, the resultant epoxy composite with 2.30 vol% of graphene exhibits an outstanding through-plane thermal conductivity of as high as 20.0 W m^−1^ K^−1^, 100 times of that of epoxy, with a record-high specific thermal conductivity enhancement of 4310%. Furthermore, the lamellar-structured graphene aerogel endows epoxy with a high fracture toughness, ~ 1.71 times of that of epoxy.

**Electronic supplementary material:**

The online version of this article (10.1007/s40820-020-00548-5) contains supplementary material, which is available to authorized users.

## Introduction

Although polymers have a wide range of applications in electronic devices, including power electronics, electric motors, and generators due to their lightweight, corrosion resistance, and ease of processing [1–4], their low thermal conductivities (~ 0.2 W m^−1^ K^−1^) severely limit their heat conduction and dissipation in electronic devices. Therefore, the improvements in thermal conductivity of polymer materials, especially their through-plane thermal conductivity, are highly crucial for their application as thermal interface materials (TIMs) between heaters and heat sinks [5]. To achieve this goal, metallic, ceramic, and carbon-based thermally conductive fillers, such as silver nanoparticles [6, 7], boron nitride nanosheets [8–10], carbon nanotubes [11], graphite [12, 13], and graphene sheets [14–17], are compounded with polymers. Among these conducting fillers, graphene becomes highly promising because of its exceptionally high in-plane thermal conductivity (~ 5300 W m^−1^ K^−1^) and mechanical properties [18]. However, dispersion of individual graphene sheets in a polymer matrix usually results in a low enhancement efficiency in thermal conductivity because of the large interface thermal resistances [19]. The thermal conductivities of polymer/graphene nanocomposites are often lower than 2 W m^−1^ K^−1^ even at the graphene loading of ~ 10 wt% [20, 21]. To obtain a high thermal conductivity over 10 W m^−1^ K^−1^, higher graphene loading is required, which would seriously deteriorate ductility and toughness of polymers [22].

To improve the enhancement efficiency in thermal conductivity, Ruoff et al. fabricated a three-dimensional (3D) graphene foam using a chemical vapor deposition (CVD) method as continuous thermal conduction paths for phase change materials, and its erythritol composite exhibited a high thermal conductivity of 3.44 W m^−1^ K^−1^ at a low graphene content of 1.23 vol% [23]. The thermal conductivity enhancement (TCE) is as high as 1800%, and the specific TCE (per 1 vol% of graphene) is about 1500%. Recently, Ren et al. used a graphene foam filled with aligned graphene sheets as a thermally conductive path, and its nature rubber composite with a graphene loading of 6.2 vol% had a high thermal conductivity of 10.64 W m^−1^ K^−1^ with its TCE of 8100% and its specific TCE of 1300% [24].

Obviously, because of the effective decreases in both the contact thermal resistance between graphene sheets and the interface thermal resistance between graphene and polymer matrix, preconstruction of a continuous graphene conduction network becomes an efficient approach for improving thermal conductivities of polymer/graphene composites [25–30]. However, the enhancement efficiency in thermal conductivity is still far from the theoretical value. It is still a great challenge for further improving the thermal conductivity enhancement efficiency of the 3D graphene network to achieve a high through-plane thermal conductivity (e.g., > 10 W m^−1^ K^−1^) at low graphene contents (e.g., < 5 wt%).

For 3D graphene aerogels used for thermally conductive polymer composites, their skeleton walls can be approximately regarded as ultrathin graphene-based films. The quality of graphene sheets and their dense compaction are crucial for heat conduction along the continuous skeletons of the aerogel. In reality, many graphene aerogels derive from GO sheets, and high temperature annealing is adopted to remove the residual oxygen-containing groups of GO sheets and heal their lattice defects to reduce the phonon scattering [31–33]. But, the gases generated during the thermal treatment could cause numerous thermally inert pores between the aligned graphene sheets, which would adversely affect the thermal conduction of the skeleton walls. As proved by Lian et al., thermal conductivity of a graphene film is closely related to its density and the annealing temperature used [34], and a porous graphene film could be readily converted to a closely stacked one by simple compression, exhibiting a high thermal conductivity of ~ 1434 W m^−1^ K^−1^ because of the significantly decreased contact thermal resistance [34]. Besides, Gao et al. reported a highly thermally conductive graphene film by using large graphene sheets for reducing lateral phonon scattering resulted from grain boundaries [35]. Near-perfect graphene crystallinity was also formed to facilitate phonon conduction, and the resultant highly aligned graphene film exhibited an outstanding thermal conductivity of ~ 2292 W m^−1^ K^−1^ [36].

Inspired by the ultrahigh thermal conductivity of densely stacked high-quality graphene films, herein, we fabricate lamellar-structured graphene aerogels (LSGAs) with continuous and highly thermally conductive paths by bidirectional freezing of a suspension of polyamic acid salt (PAAS) and graphene oxide (GO), followed by lyophilization, imidization, and graphitization treatments. By regulating polyamic acid (PAA)/GO mass ratios, an optimal lamellar architecture is achieved during the bidirectional freezing process, where PAAS and GO components are expelled by bidirectionally grown ice crystals to form the numerous vertically aligned lamellae. By subsequent imidization treatments at 300 °C, the PAAS monomers are polymerized to polyimide (PI) macromolecules, while the GO is thermally reduced to RGO by partially removing its residual oxygen-containing groups. Finally, graphitization at 2800 °C is adopted to graphitize PI macromolecules to be graphitized carbon with the help of the induced orientation effect of RGO and simultaneously upgrade RGO to be high-quality graphene with negligible lattice defects and large crystal sizes. The resultant lamellar-structured aerogel possesses superior through-plane thermal conduction capacity because of its vertically aligned and closely stacked high-quality graphene lamellae. Besides, the conventional graphene aerogels are proud of their low apparent density [37, 38], which is not conducive to endowing polymers with high thermal conductivity. To address this issue, the LSGAs infiltrated with epoxy monomer and curing agents are compressed slowly along the direction perpendicular to the lamellar surface. Thanks to the high compressibility of LSGAs [39], their apparent densities could be tuned by varying the compression extents without damaging the lamellar structures. The nacre-like graphene/epoxy composite exhibits an outstanding through-plane thermal conductivity of ~ 20.0 W m^−1^ K^−1^ at a low graphene loading of ~ 2.30 vol%, as well as a high TCE of ~ 9915% and a record-high specific TCE of ~ 4310%. Furthermore, the lamellar structure of the LSGA endows its thermally conductive nacre-like epoxy composite with high fracture toughness.

## Experimental Section

### Materials

1,2-Bis(2,3-epoxypropoxy)ethane as the reactive diluent, methyl hexahydrophthalic anhydride as the curing agent, and 2,4,6-tris(dimethylaminomethyl)phenol as the curing accelerator were purchased from Adamas Reagents (China). Bisphenol-A epoxy resin was supplied by Jiafa Chemicals (China). Sulfuric acid (98%), hydrochloric acid (37%), hydrogen peroxide (30%), and potassium permanganate (99.5%) were supplied by Beijing Chemical Reagents (China). Pyromellitic dianhydride (PMDA, 99.0%), 4,4′-diaminodiphenyl ether (ODA, 99.0%), N,N-dimethylacetamide (DMAc), triethylamine (TEA), and sodium nitrate were purchased from Aladdin (China). Natural graphite flakes (300 meshes) were purchased from Huatai Lubricant & Sealing (China). All chemicals and reagents were used as received without further purification.

### Synthesis of Graphene Oxide and Polyamic Acid

Graphite oxide was synthesized by oxidizing graphite flakes with a modified Hummers method [40]. GO sheets with average lateral size of ~ 1 μm and thickness of ~ 1 nm (Fig. S1) were obtained by ultrasonic exfoliation of graphite oxide with an ultrasonicator at a power of 270 W. Polyamic acid (PAA) was synthesized using a method reported in our previous work [41]. Typically, DMAc (200 mL) was added into a 500-mL three-neck round bottom flask fitted with a mechanical stirrer, ice bath, and nitrogen gas protection, and ODA (5.71 g) was then added. Once the ODA was dissolved completely, PMDA (6.28 g) was added into the solution in 60 min, and the mixture was stirred for 5 h at 0 °C. The obtained viscous solution was slowly poured into deionized water, and the resultant precipitate was washed with deionized water and vacuum-dried at 50 °C for 48 h.

### Fabrication of Lamellar-Structured PAAS/GO Hybrid Aerogels

**Fig. 1:**
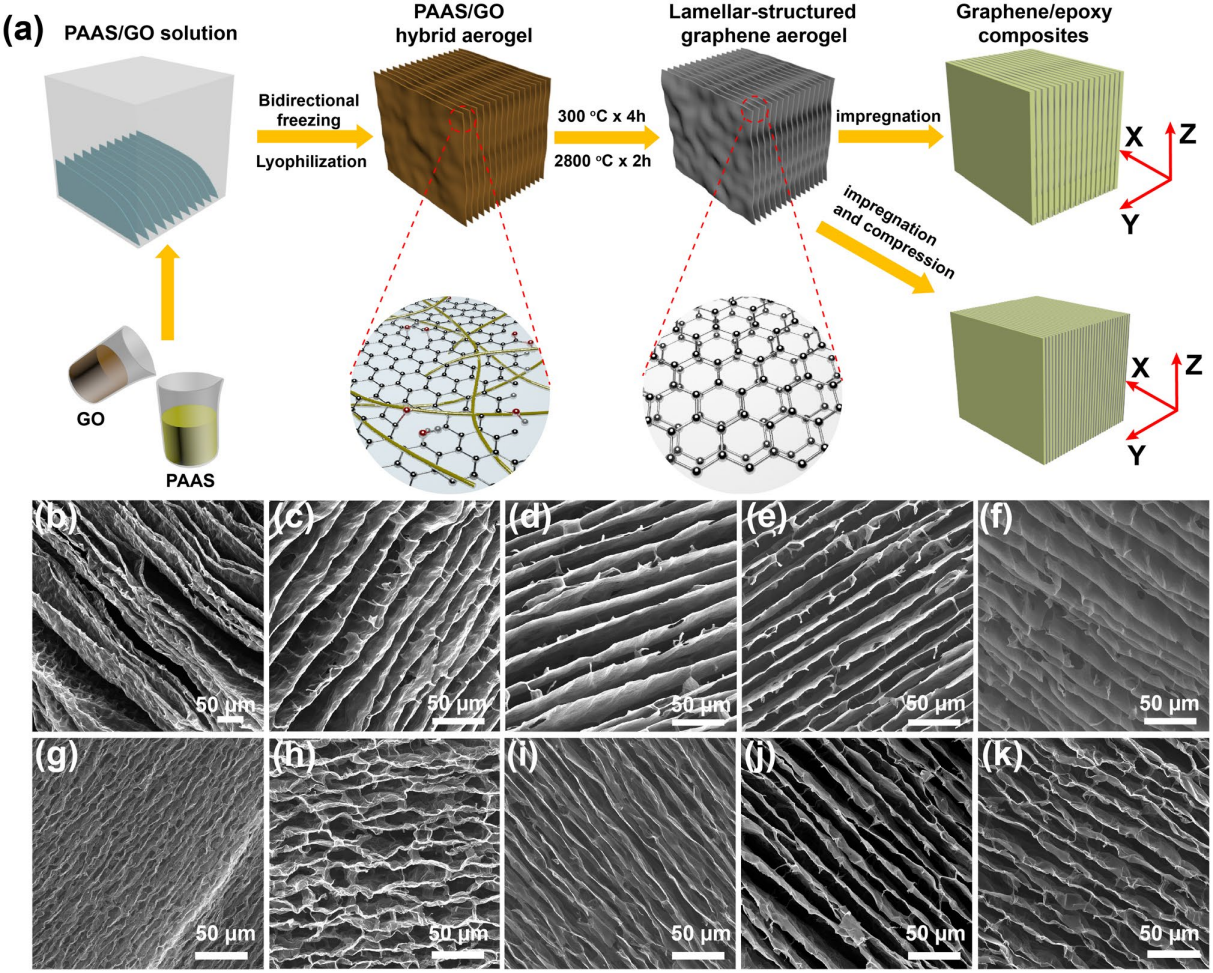
**a** Schematic illustration of fabrication of a LSGA and its epoxy composite. SEM images of morphologies of **b** P9G1, **c** P8G2, **d** P7G3, **e** P6G4, and **f** P5G5 observed along Z-axis; SEM images of morphologies of **g** P9G1-2800, **h** P8G2-2800, **i** P7G3-2800, **j** P6G4-2800, and **k** P5G5-2800 observed along Z-axis

PAAS was prepared by dissolving PAA and TEA in deionized water under magnetic stirring for 2 h with a mass ratio of 1:0.48. After a GO suspension was added into the PAAS solution, the obtained PAAS/GO suspension was sonicated for 15 min and magnetically stirred for 2 h. The initial PAA/GO mass ratio varied from 9:1 to 3:7 to obtain different PAAS/GO suspensions, but the total concentration of PAAS/GO suspensions is kept at ~ 4 wt%. PAAS/GO hybrid aerogels were prepared by bidirectional freezing and lyophilization (Fig. 1a). Briefly, a PAAS/GO suspension was transferred into a rectangular silicone mold (20 × 20 × 20 mm^3^ or 30 × 50 × 20 mm^3^) that placed on a copper bridge, and then, one end of the copper bridge was inserted into liquid nitrogen to form a bidirectional temperature gradient. The frozen suspension was freeze-dried in a freeze-dryer (− 50 °C, < 10 Pa) for 72 h. The resultant PAAS/GO hybrid aerogel was designated as PxGy, where x:y is the PAA/GO mass ratio. For comparison, neat PAAS aerogel and GO aerogel were also prepared using the same methodology. Detailed ingredients for the preparation of PAAS/GO hybrid aerogels are provided in Table S1.

### Preparation of High-Quality Graphene Aerogels

The LSGAs were prepared by imidization and graphitization of PAAS/GO hybrid aerogels. The obtained PAAS/GO hybrid aerogels were thermally annealed at 300 °C for 4 h under an argon atmosphere to polymerize PAAS to PI macromolecules and to thermally reduce GO to RGO. Subsequently, the PI/RGO aerogels were graphitized at 2800 °C for 2 h to obtain the lamellar-structured high-quality graphene aerogels, which were designated as GxPy-2800, where x:y is the initial mass ratio of a PAA/GO suspension. For comparison, an isotropic graphene aerogel (IP6G4-2800) was prepared by conventional freezing the PAAS/GO suspension in liquid nitrogen followed by freeze-drying, imidization, and graphitization. A unidirectionally orientated graphene aerogel (UP6G4-2800) was also fabricated by unidirectional freezing the PAAS/GO suspension followed by freeze-drying, imidization, and graphitization.

### Fabrication of Thermally Conductive Graphene/Epoxy Composites

The graphene/epoxy composites (GEs) were fabricated by vacuum-assisted impregnation followed by thermal curing. Epoxy resin, the reactive diluent, the curing agent, and the curing accelerator were mixed uniformly with the mass ratio of 8:2:9.48:0.0576, and then, the LSGAs were immersed into the mixture under vacuum for 12 h, followed by curing at 80 °C for 4 h and post-curing at 120 °C for 2 h. The resultant composites were designated as GEx, where x varies from 1 to 5, representing the initial mass ratio of PAA to GO from 9:1 to 5:5. To increase the graphene content of the resultant composites, the LSGA (P6G4-2800) was compressed perpendicular to the lamellar surface before epoxy resin was thermally cured. The obtained composites were designated as GE4-y, where y is the compression extents, which can be 30%, 50%, and 70%. As a reference, the IP6G4-2800/epoxy composites and UP6G4-2800/epoxy composites were also prepared using the similar infiltration and curing processes and designated as IGE4 and UGE4, respectively.

### Characterization

The morphologies of PAAS/GO hybrid aerogels, LSGAs, and GEs were characterized with a Hitachi S4700 scanning electron microscope (SEM). X-ray diffraction (XRD) patterns were recorded using a Rigaku D/Max 2500 X-ray diffractometer at a generator voltage of 40 kV. A Renishaw inVia Raman microscope at an excitation wavelength of 514 nm was used to obtain Raman mapping images. An area of 40 × 40 μm^2^ was automatically scanned with a XY stage using a step size of ~ 1 μm, and Raman spectra were recorded at every points. The crystal sizes of LSGAs were calculated with the empirical formula: La(nm) = (2.4 × 10^−10^)*λ*^4^(*I*_D_/*I*_G_)^−1^ [42], where λ is the laser wavelength, and *I*_D_/*I*_G_ is the integrated intensity ratio of D band to G band. Thermal conductivities were calculated by *k* = *α* × *ρ*×*C*_p_, where *α* is the thermal diffusivity, *C*_p_ is the specific heat capacity, and *ρ* is the density. Thermal diffusivities of GEs were measured on a Netzsch LFA467 light flash apparatus from 30 to 80 °C. Specific heat capacities of GEs from 30 to 80 °C were obtained using a TA Q20 differential scanning calorimeter (DSC) at a scanning rate of 10 °C min^−1^. Densities of GEs were measured by an Mettler Toledo electronic balance with a density determination kit 33,360. Thermogravimetric analysis (TGA) curves were obtained with a TA Q50 thermogravimetric analyzer at a heating rate of ~ 10 °C min^−1^ in a nitrogen atmosphere. The volume fraction was calculated by: vol% = wt% × (*ρ*_com_/ρ*), where wt% is the mass fraction, *ρ*_com_ is the density of a composite, and ρ* is the true density of graphene (2.25 g cm^−3^). The heat transfer performances of GEs were recorded by a FLIR E40 infrared camera. High-resolution transmission electron microscopy (HRTEM) images were obtained using a JEM-2100Plus microscope at an operation voltage of ~ 200 kV. Three-point bending tests (span 7.75 mm) were performed on a SUNS UTM4103 tester with a loading rate of 0.05 mm min^−1^. The loading direction is perpendicular to the lamellar surface of the LSGA. For single-edge notched beam specimens, the epoxy and GEs were cut and polished to beams with dimension of 12 × 2.5 × 2.5 mm^3^, and the beams were then notched by a diamond blade. The notch was sharpened by a razor blade perpendicular to the lamellar direction. The notch was about half of the thickness of specimens. The initial fracture toughness (*K*_IC_) was calculated by Eq. (1) [43]:

1 $$K_{IC} = \frac{{P_{IC} S}}{{BW^{3/2} }}f\left( {\frac{a}{W}} \right),\,\,f\left( {\frac{a}{W}} \right) = \frac{{3\left( {\frac{a}{W}} \right)^{1/2} \left[ {1.99 - \frac{a}{W}\left( {1 - \frac{a}{W}} \right)\left( {2.15 - 3.93\frac{a}{W} + 2.7(a/W)^{2} } \right)} \right]}}{{2\left( {1 + 2\frac{a}{W}} \right)\left( {1 - \frac{a}{W}} \right)^{3/2} }}$$

where *P*_IC_ is the maximum load before crack initiation, *B* is the width, *S* is the span, *W* is the thickness, and *a* is the notch depth of the specimens. The maximum fracture toughness (*K*_J_) was calculated by Eq. (2) [44]:

2 $$K_{J} = \sqrt {\frac{{EJ_{PI} }}{{1 - \nu^{2} }} + K_{IC}^{2} } ,\,\,J_{PI} = \frac{{2A_{PI} }}{{B\left( {W - a} \right)}}.$$

where *A*_PI_ is the area under the force–displacement curve, E is the Young’s modulus, and ν is the Poisson’s ratio. The crack extension (Δa) is calculated by Eq. (3) [44]:

3 $$a_{n} = a_{n - 1} + \frac{{W - a_{n - 1} }}{2}\frac{{C_{n} - C_{n - 1} }}{{C_{n} }},\,\,C_{n} = \frac{{u_{n} }}{{f_{n} }},\,\,{{\Delta a}} = a_{n} - a$$

where $a_{n}$ is the crack length, $u_{n}$ is the displacement, and $f_{n}$ is the force at each point after crack initiation.

## Results and Discussion

### Morphologies and Microstructures of Lamellar-Structured Graphene Aerogels

Figure 1a illustrates the fabrication of LSGAs and their epoxy composites. During the bidirectional freezing of the PAAS/GO suspension, ice crystals nucleate and grow to be parallel lamellae because of the temperature gradients in both horizontal and vertical directions, expelling the PAAS and GO components from the ice crystal lamellae to replicate the lamellar morphology [45, 46]. By freeze-drying to remove the ice crystals by their subliming, the resultant PAAS/GO hybrid aerogels are thermally annealed at 300 °C, during which the PAAS monomers are polymerized to PI, while the GO component is partially reduced to RGO. The bidirectionally orientated PI/RGO hybrid aerogel is less thermally conductive because of the less conductive PI and the poor conductivity of RGO. Therefore, the resultant PI/RGO hybrid aerogel is graphitized at 2800 °C to carbonize and even graphitize the thermally insulating PI macromolecules and to convert RGO to high-quality graphene by removing its residual oxygen-containing groups and healing its lattice defects.

As expected, the PAAS/GO hybrid aerogels show lamellar and porous structures, and the spacing between adjacent lamellae varies in the range of 20-40 μm (Fig. 1b–f). It is also seen that the initial dosage of GO greatly affects the lamellar structure of the hybrid aerogels, especially when the dosage of GO exceeds 50 wt%. As shown in Fig. S2, P6G4 and P5G5 have long-range lamellar structures (Fig. S2a, b). Further increasing the GO dosage tends to form disordered structures (Fig. S2c, d). Particularly, a neat GO aerogel derived from a concentrated GO suspension (40 mg cm^−3^) in the absence of PAAS presents a completely disordered structure (Fig. S2e, j). This is not only because the oxygen-containing groups on the GO sheets are willing to be adsorbed to the surface of the ice crystal, and hence, causing a curved ice crystal during freezing process, the high viscosity of GO suspension at such a high concentration also hinders the unidirectional growth of ice crystal [47]. Fortunately, after the imidization and graphitization treatments, the anisotropic LSGAs still maintain their lamellar structures (Fig. 1g–k). The bidirectional orientation extents of the lamellae are affected by initial PAA/GO mass ratios. P9G1-2800 and P8G2-2800 show a waved multi-arch morphology. With increasing the GO dosage, the lamellae change from crumpled to relative flat. This is because the volume decrease in PI component during the graphitization process is larger than that of GO sheets, giving rise to uneven stress distribution in the lamellae, especially at high PI contents [39]. Consequently, a lamellar-structured high-quality graphene aerogel is thus fabricated (Fig. S3).

**Fig. 2:**
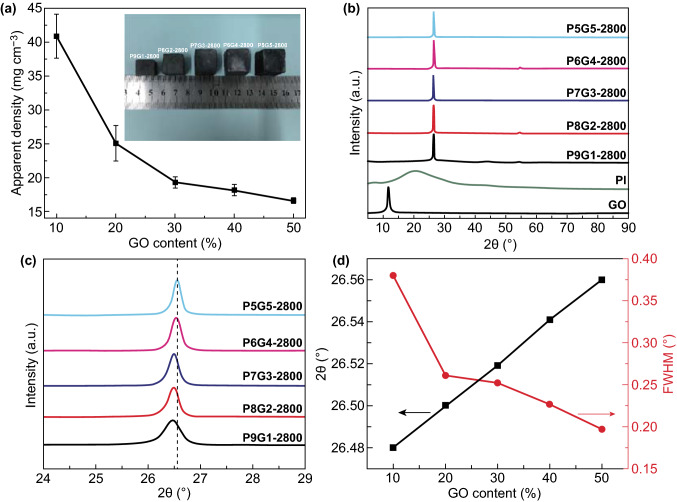
**a** Plots of apparent density of LSGAs versus GO content in PAAS/GO suspensions; the inset shows the different sizes of LSGAs after the graphitization treatment. **b, c** XRD patterns of GO, PI, and LSGAs. **d** Plots of (002) diffraction angle and FWHM of LSGAs as a function of GO content in PAAS/GO suspensions

For graphene aerogels used as thermally conductive fillers, their apparent density determines the graphene content in composites, which affects the thermal conductivities of resultant composites. Here, because the volume decrease in GO is less than that of PI during their graphitization process, the apparent density of LSGAs decreases from ~ 40.9 to ~ 16.6 mg cm^−3^ as the GO content increases from 10 to 50 wt% (Fig. 2a). In addition to the apparent density of the LSGAs, the graphene quality is also crucial for efficient heat conduction along the aerogel skeleton walls. XRD is used to characterize the graphene quality by probing the amount and orientation of graphitic carbon layers and the curvature of individual sheets [48]. As shown in Fig. 2b, GO shows a sharp peak at ~ 11.7°, while PI exhibits a broad peak at ~ 21.1° due to its semicrystalline nature. Interestingly, after the high-temperature graphitization, all the resulting LSGAs exhibit sharp peaks. The peak position shifts from 26.48° for P9G1-2800 to 26.56° for P5G5-2800, and the full width at half maximum (FWHM) decreases from 0.38° of P9G1-2800 to 0.20° of P5G5-2800 simultaneously (Fig. 2c, d). These results prove that the increase in the GO dosage results in increases in graphene crystallinity as well as decreases in graphene sheet curvature [48, 49].

**Fig. 3:**
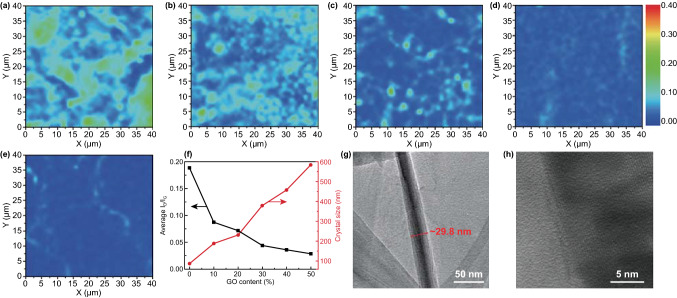
Raman mappings of **a** G9P1-2800, **b** G8P2-2800, **c** G7P3-2800, **d** G6P4-2800, and **e** G5P5-2800. **f** Plots of average I_D_/I_G_ value and crystal size of LSGAs as a function of GO content in PAAS/GO suspensions. **g** TEM and **h** HRTEM images of G6P4-2800

The graphene quality in LSGAs is further evaluated with Raman mappings by integrated intensity ratios (*I*_D_/*I*_G_) of D band (~ 1350 cm^−1^) and G band (~ 1580 cm^−1^) [50]. Different from Raman spectrum at single points, Raman mapping reflects the distribution of lattice defects more accurately in a certain area. As shown in Fig. 3a–e, different colors represent different *I*_D_/*I*_G_ ratios. The blue, green, and red colors correspond to their *I*_D_/*I*_G_ values of 0, ~ 0.2, and ~ 0.4, respectively. Compared to PAA-2800 with an average *I*_D_/*I*_G_ of ~ 0.189 (Fig. S4a), the graphitization extent of P9G1-2800 is greatly improved with a low average *I*_D_/*I*_G_ of ~ 0.087, although it still presents large green and glaucous areas. Interestingly, as the increase in the GO dosage, the blue area becomes larger, while the green area decreases gradually. The average *I*_D_/*I*_G_ decreases to ~ 0.028 for P5G5-2800, very close to that of GO-2800 (~ 0.026) (Fig. S4b). Meanwhile, as shown in Fig. 3f, the decreased intensity of *I*_D_/*I*_G_ manifests the stepwise healing of *sp*^2^ domains from ~ 87.4 nm of PAA-2800 to ~ 188.9 nm of P9G1-2800, and then to ~ 583.4 nm of P5G5-2800 [42]. Apparently, these Raman mappings indicate that graphitization converts PI to graphitized carbon and reduce GO to high-quality graphene. It is also seen that GO plays an inducing role in the conversion of PI to graphitized carbon because the large sheet of GO could promote the orientation of PI macromolecules and thus improve the graphitization extent of LSGAs [51, 52].

In addition to the low lattice defects, and large graphene crystal size, the high quality of the vertically aligned skeleton walls is also crucial for thermal conduction of LSGAs. HRTEM is adopted to observe the skeleton wall of P6G4-2800 (Fig. 3g, h). The lamella thickness is ~ 29.8 nm (Fig. 3g), and the P6G4-2800 possesses vertically aligned and closely stacked graphitic lamellae, which is similar to a highly thermally conductive graphitic film [36]. The closely stacked skeleton walls of P6G4-2800 would benefit the decrease in contact thermal resistances. It is believed the PAAS joints and fills the gaps between the GO sheets and both of them are expelled to form vertically aligned and closely stacked lamellae by generated ice crystals during the bidirectional freezing, and the closely stacked lamellae are converted to orderly stacked graphitic layers after the imidization and graphitization treatments (Fig. 3g) [53]. Moreover, due to the ultrathin lamellae and the highly porous structure of the PAAS/GO hybrid aerogel, the gases released during the imidization and graphitization process may escape easily, which would not cause the gases accumulation and the froth of the closely stacked lamellae [34, 35]. More importantly, as the PAAS component is approximately continuous between GO sheets in the lamellae of a PAAS/GO aerogel, its graphitic counterpart can be regarded as ultra-large graphene sheets, which would benefit the growth of graphene crystals with nearly perfect in-plane crystallinity (Fig. 3f) to reduce the lateral phonon scattering [54]. The π–π interaction between the high-quality graphene sheets also facilitates the close stacking of the lamellae during the graphitization process. All these results prove that LSGAs are highly promising for thermal conduction applications.

### Thermally Conductive Properties of LSGA/Epoxy Composites

**Fig. 4:**
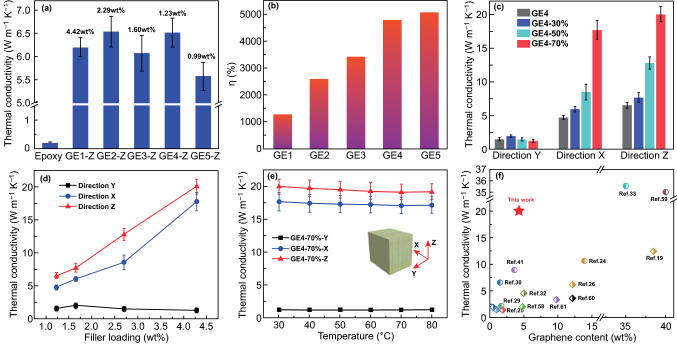
**a** Thermal conductivities along Z-direction, and **b** specific TCEs of graphene/epoxy composites. The data in **a** are graphene contents in their epoxy composites. **c** Comparison of thermal conductivities of GE4, GE4-30%, GE4-50%, and GE4-70% in three directions. **d** Plots of thermal conductivity of the composites in three directions as a function of graphene content. **e** Thermal conductivities of GE4-70% in three directions at different temperatures. **f** Comparison of thermal conductivity of GE4-70% in Z-direction with those reported in the literature

By a vacuum-assisted filtration of LSGAs with epoxy monomer and curing agents followed by thermal curing, the resultant LSGA/epoxy composites still show a nacre-like structure (Fig. S5a–c). Due to the anisotropic lamellar structure of LSGAs, their composites exhibit different thermal conductivities in three directions, and the thermal conductivity in Z-direction is higher than those along the other two directions (Fig. S6a) [55]. The through-plane thermal conductivities (Z-direction) of the composites are much higher than that of neat epoxy (~ 0.20 W m^−1^K^−1^) (Fig. 4a). Moreover, compared to the isotropic graphene network (IP6G4-2800) (Fig. S7a, c) and the unidirectionally orientated graphene network (UP6G4-2800) (Fig. S7b, d), the lamellar-structured graphene network endows epoxy with a higher through-plane thermal conductivity (Fig. S6b). More importantly, the XRD patterns (Fig. S8a, b) as well as the Raman mappings (Fig. S8c, d) of IP6G4-2800 and UP6G4-2800 show that both of them possess the same high-quality graphene as P6G4-2800, further indicating the superior thermal conduction of the lamellar structure along Z-direction [56]. Table S2 lists through-plane thermal conductivities of the LSGA/epoxy composites, the average I_D_/I_G_ values of LSGAs, and the graphene contents calculated on the basis of the TGA curves (Fig. S9). As shown in Fig. 4a and Table S2, both the quality of LSGAs and the graphene content affect the ultimate thermal conductivity of the epoxy composites. To eliminate the effect of graphene content and highlight the role of the quality of LSGAs, the efficiency of thermal conductivity enhancement (η) is regarded as specific TCE and calculated by Eq. (4):

4 $$\eta = \left[ {\left( {K - K_{m} } \right)/\left( {100 \, VK_{m} } \right)} \right] \times 100\%$$

where *K*_m_ (W m^−1^ K^−1^) and *K* (W m^−1^ K^−1^) are thermal conductivities of epoxy and LSGA/epoxy composites, respectively, and *V* (vol%) is the volume fraction of fillers. As shown in Fig. 4b, GE5 has the highest specific TCE of ~ 5054%, resulting from the highly efficient thermal conduction path of P5G5-2800. Compared to other composites shown in Fig. 4a, GE4 exhibits the highest thermal conductivity of ~ 6.51 W m^−1^ K^−1^ with a relatively high specific TCE of ~ 4750% at the graphene content of ~ 1.23 wt%, because GE4 owns highly efficient thermal conduction paths as well as relatively high filler content.

To further improve thermal conductivity of the epoxy composites while maintaining their mechanical properties, the graphene content can be increased by slowly compressing the LSGAs perpendicular to the lamellar direction before the epoxy resin is thermally cured. As shown in Fig. 4c, d, the compression extents of P6G4-2800 can be 30%, 50%, and 70%, and the graphene contents in the epoxy composites increase from ~ 1.23 wt% of GE4 to ~ 4.28 wt% of GE4-70%. Meanwhile, the thermal conductivities in Z- and X-directions of the composites enhance with increasing the compression extents. For example, the thermal conductivity of GE4 along Z-direction is ~ 6.51 W m^−1^ K^−1^, while that of GE4-70% increases to ~ 20.0 W m^−1^ K^−1^, because the lamellar sheets become more compact, while the nacre-like structure of the composites is well retained (Fig. S5d-f) [57]. Note that the thermal conductivities do not change significantly in Y-direction with increasing the graphene content. As shown in Fig. 4d, the thermal conductivity of GE4-70% in Z-direction is ~ 20.0 W m^−1^ K^−1^, while that of GE4-70% in Y-direction is only ~ 1.22 W m^−1^ K^−1^, which is ascribed to the high interface thermal resistances between graphene and polymer matrix in the conduction path of the Y-direction.

The influence of temperature on thermal conductivity of the thermosetting epoxy composites is also evaluated (Fig. 4e). When the temperature increases from 30 to 80 °C, the thermal conductivity of GE4-70% decreases slightly in three directions. Nevertheless, the through-plane thermal conductivity (Z-direction) of GE4-70% at 80 °C is still as high as ~ 19.2 W m^−1^ K^−1^. As shown in Fig. 4f, the GE4-70% with ~ 4.28 wt% (2.30 vol%) of graphene exhibits a high through-plane thermal conductivity of ~ 20.0 W m^−1^ K^−1^, much higher than those reported in the literature at similar graphene contents [14, 19, 24–26, 28–30, 32, 33, 41, 58–61]. Moreover, GE4-70% presents a record-high specific TCE of ~ 4310% at such a high thermal conductivity among all kinds of fillers, which is even higher than those of film-type composites that usually have high in-plane thermal conductivities (Table S3) [24, 27, 59, 62]. Due to the superior elasticity of the lamellar structure, the graphene content could be tuned by compression along Z-direction without damaging the closely stacked graphene lamellae, and the enhancement efficiency only decreases a little when the graphene content increases from ~ 1.23 to ~ 4.28 wt%.

**Fig. 5:**
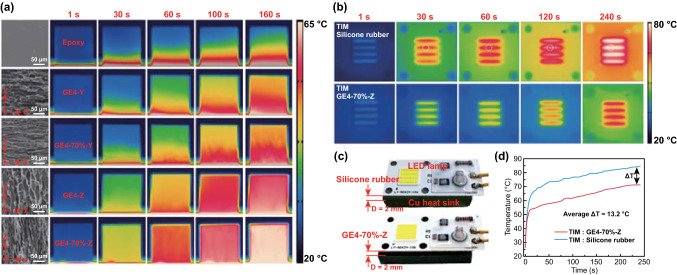
**a** Infrared images of epoxy and its composites on the same hot stage at 75 °C, showing that GE4-70%-Z has the best thermal conduction efficiency. SEM images in the left column show the morphologies of epoxy and composites. **b** Top-view infrared images of the LED chips during working, indicating more efficient heat dissipation when GE4-70%-Z is used as TIM. **c** Digital photographs showing two LED chips integrated with commercial silicone rubber and GE4-70%-Z as TIMs. **d** Comparison between the temperature increases in the same plot on two chips, depicted by the white dotted circle in **b**

To illustrate the difference in thermal conductivities intuitively, the epoxy and its composites with a dimension of 10 × 10 × 10 mm^3^ are placed on the same hot stage at 75 °C, and an infrared camera is adopted to record in situ the side temperature variation of the samples (Fig. 5a, Movie S1). Figure 5a shows the infrared images after heating for 1, 30, 60, 100, and 160 s. The side temperature of GE4-70% along Z-direction (GE4-70%-Z) increases much faster than others, which should be attributed to its high through-plane thermal conductivity resulted from the high-quality vertically orientated graphene and the high graphene content of 4.28 wt%. The GE4-70%-Z is highly promising as TIM because of its exceptional high through-plane thermal conductivity. As shown in Fig. 5c, commercial silicone rubber with a thermal conductivity of ~ 6 W m^−1^ K^−1^ and GE4-70%-Z are inserted between a 10 W LED chip and a Cu heat disk. The thickness of the TIM is ~ 2 mm, and the LED chip/TIM/Cu heat disk interfaces are glued by a thermally conductive silicone grease. The surface temperatures of the LED chips are recorded with an infrared camera upon lightening (Fig. 5b). The series of infrared images reveal that the temperature increases sharply with the silicone rubber as TIM as compared to GE4-70%-Z. With the commercial silicone rubber as the TIM, the final surface temperature is up to ~ 84.5 °C, whereas the temperature is only ~ 71.3 °C in the presence of GE4-70%-Z (Fig. 5d) because of the excellent heat dissipation ability of GE4-70% along the Z-direction.

### Fracture Behavior of Epoxy and LSGA/Epoxy Composites

**Fig. 6:**
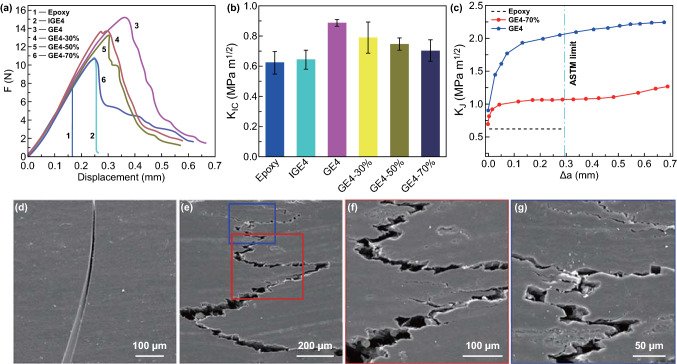
**a** Typical force–displacement curves of epoxy, IGE4, GE4, GE4-30%, GE4-50%, and GE4-70%. **b** K_IC_ comparison of epoxy and our graphene/epoxy composites. **c** Rising R curve of maximum fracture toughness versus the crack length. **d** SEM images show the straight crack propagation. **e–g** SEM images show the tortuous crack growth of GE4-70%; **f** and **g** are enlarged versions of the selected part in **e**

Apart from the high through-plane thermal conductivity, the nacre-like structure also endows the composites with high fracture toughness. The typical force–displacement curves (Fig. 6a) show that epoxy is brittle (curve 1). Because of the enhancement of graphene networks, the force–displacement curve of IGE4 (composite with an isotropic graphene network) is higher than that of epoxy, but is still brittle (curve 2) [63]. The force–displacement curve of GE4 is much higher than those of epoxy and IGE4. With increasing the compression extent, the force–displacement curves show decreases (curves 3–6), but are still higher than those of epoxy and IGE4. Figure 6b shows the initial fracture toughness (*K*_IC_) results calculated on the basis of the force–displacement curves. *K*_IC_ of the IGE4 is ~ 0.64 MPa m^1/2^, slightly higher than that of epoxy (~ 0.62 MPa m^1/2^). Fortunately, the lamellar-structured GE4 exhibits an enhanced fracture toughness of ~ 0.88 MPa m^1/2^, ~ 1.38-fold that of the IGE4 at the same graphene content. Consistent with the force–displacement curves, the K_IC_ decreases gradually to ~ 0.70 MPa m^1/2^ when the compression extent increases to 70%. As reported previously, when the graphene content is beyond a certain value, the fracture toughness of its epoxy composite would decrease [64]. Fortunately, as compared to neat epoxy, the lamellar structure of LSGA endows its epoxy composite with a high through-plane thermal conductivity of ~ 20 W m^−1^ K^−1^ as well as a high fracture toughness at a relatively low graphene content. As shown in Fig. 6c, rising resistance curves (R curve) are calculated to explain the toughness of GE4 and GE4-70%. As the cracks continue to grow, the maximum fracture toughness (*K*_J_) of GE4 gradually increases to ~ 2.06 MPa m^1/2^ within the American Society for Testing and Materials (ASTM) limit (E1820-13) [65], which is ~ 3.32-fold that of neat epoxy. Although the K_J_ of GE4-70% is lower than that of GE4, it still reaches ~ 1.06 MPa m^1/2^, which is ~ 1.71-fold that of epoxy.

Crack propagation is used to explain the high fracture toughness of GE4-70% (Fig. 6d–g). The crack of epoxy is straight along the tip of the notch (Fig. 6d). In contrast, the crack propagation of GE4-70% is tortuous as shown in Fig. 6e. The enlarged images (Fig. 6f, g) show that the crack deflection, crack branching, and interfacial friction during the crack propagation dissipate a large amount of energy and sequentially endow GE4 and GE4-70% with high fracture toughness [43, 44, 66]. The fracture morphologies of epoxy and GE4-70% illustrate that the graphene lamellae are debonded and even pulled out from the epoxy matrix (Fig. S10), which also contributes to the enhancement in fracture toughness.

## Conclusions

Lamellar-structured PAAS/GO aerogels are fabricated by bidirectional freezing of PAAS/GO suspensions followed by lyophilization and converted to PI/RGO aerogels by thermal treatment at 300 °C, during which PAAS monomers are polymerized to PI macromolecules, while GO is thermally reduced to RGO. The final graphitization at 2800 °C is crucial for obtaining the lamellar-structured high-quality graphene aerogels, during which PI is carbonized and even graphitized to thermally conductive carbon with the inductive effect of RGO, while RGO is simultaneously upgraded to high-quality graphene by thermally removing its residual oxygen-containing groups and healing its lattice defects. By adjusting the initial mass ratio of PAA and GO, an optimal LSGA with superior thermally conductive capacity is obtained because of its continuous network, densely stacked graphene lamellae, and large graphene sizes. Thanks to the excellent compressibility, the lamellar-structured graphene aerogel infiltrated with epoxy monomer and curing agent could be compressed perpendicular to the lamellar direction to adjust the graphene content in the resultant graphene/epoxy composite. The nacre-like anisotropic composite exhibits different thermal conductivities along three directions, and its through-plane thermal conductivity can be as high as ~ 20.0 W m^−1^ K^−1^ at a low graphene content of ~ 2.30 vol%, with a high TCE of ~ 9915% and a record-high specific TCE of ~ 4310%. In addition, the lamellar-structured graphene aerogel also endows epoxy with an enhanced fracture toughness. Our nacre-like graphene/epoxy composite with high through-plane thermal conductivity and fracture toughness demonstrates an insightful avenue for fabrication of high-performance thermal interface materials.

## Electronic supplementary material

Below is the link to the electronic supplementary material.

SEM and AFM images of GO; SEM images of PAAS/GO hybrid aerogels and GO aerogel; SEM images of P6G4-2800; Raman mapping images of PAA-2800 and GO-2800; SEM images of composites; comparison of thermal conductivities of GE4; comparison of thermal conductivities of composites; SEM images and Raman mappings of IP6G4-2800 and UP6G4-2800; XRD patterns of P6G4-2800, IP6G4-2800, and UP6G4-2800; TGA curves of epoxy and its composites; fracture surfaces of epoxy and GE4-70%; detailed ingredients of PAAS/GO hybrid aerogels; filler contents, through-plane thermal conductivities of composites, and average I_D_/I_G_ values of LSGAs; and comparison of thermal conductivities and specific TCE of composites with those reported. (DOC 18656 kb)

Supplementary material 2 (MP4 127 kb)
