# Supplementary material for: 3D Lamellar-Structured Graphene Aerogels for Thermal Interface Composites with High Through-Plane Thermal Conductivity and Fracture Toughness
Source: Nanomicro Lett. 2020 Nov 11;13:22. doi: 10.1007/s40820-020-00548-5 (PMC8187529; doi:10.1007/s40820-020-00548-5)
Supplement: Supplementary file 1 — SEM and AFM images of GO; SEM images of PAAS/GO hybrid aerogels and GO aerogel; SEM images of P6G4-2800; Raman mapping images of PAA-2800 and GO-2800; SEM images of composites; comparison of thermal conductivities of GE4; comparison of thermal conductivities of composites; SEM images and Raman mappings of IP6G4-2800 and UP6G4-2800; XRD patterns of P6G4-2800, IP6G4-2800, and UP6G4-2800; TGA curves of epoxy and its composites; fracture surfaces of epoxy and GE4-70%; detailed ingredients of PAAS/GO hybrid aerogels; filler contents, through-plane thermal conductivities of composites, and average ID/IG values of LSGAs; and comparison of thermal conductivities and specific TCE of composites with those reported. (DOC 18656 kb) [file 40820_2020_548_MOESM1_ESM.doc]

Supporting Information for

**3D Lamellar-Structured Graphene Aerogels for Thermal Interface Composites with High Through-Plane Thermal Conductivity and Fracture Toughness**

Pengfei Liu^1, 2^, Xiaofeng Li^1,*^, Peng Min^1^, Xiyuan Chang^2^, Chao Shu^1^, Yun Ding^1^, Zhong-Zhen Yu^2, 3,*^

^1^ Beijing Key Laboratory of Advanced Functional Polymer Composites, Beijing University of Chemical Technology, Beijing 100029, China

^2^ State Key Laboratory of Organic-Inorganic Composites, College of Materials Science and Engineering, Beijing University of Chemical Technology, Beijing 100029, China

^3^ Beijing Advanced Innovation Center for Soft Matter Science and Engineering, Beijing University of Chemical Technology, Beijing 100029, China

*Corresponding authors. E-mail: [xfli@mail.buct.edu.cn](mailto:xfli@mail.buct.edu.cn) (X. Li); [yuzz@mail.buct.edu.cn](mailto:yuzz@mail.buct.edu.cn) (Z.-Z. Yu)

**Supplementary Figures and Tables**


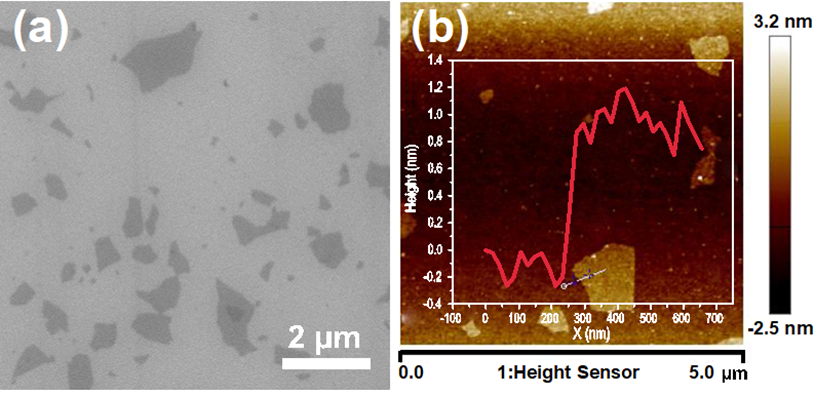


**Fig. S1.** (a) SEM image and (b) AFM image of GO sheets.


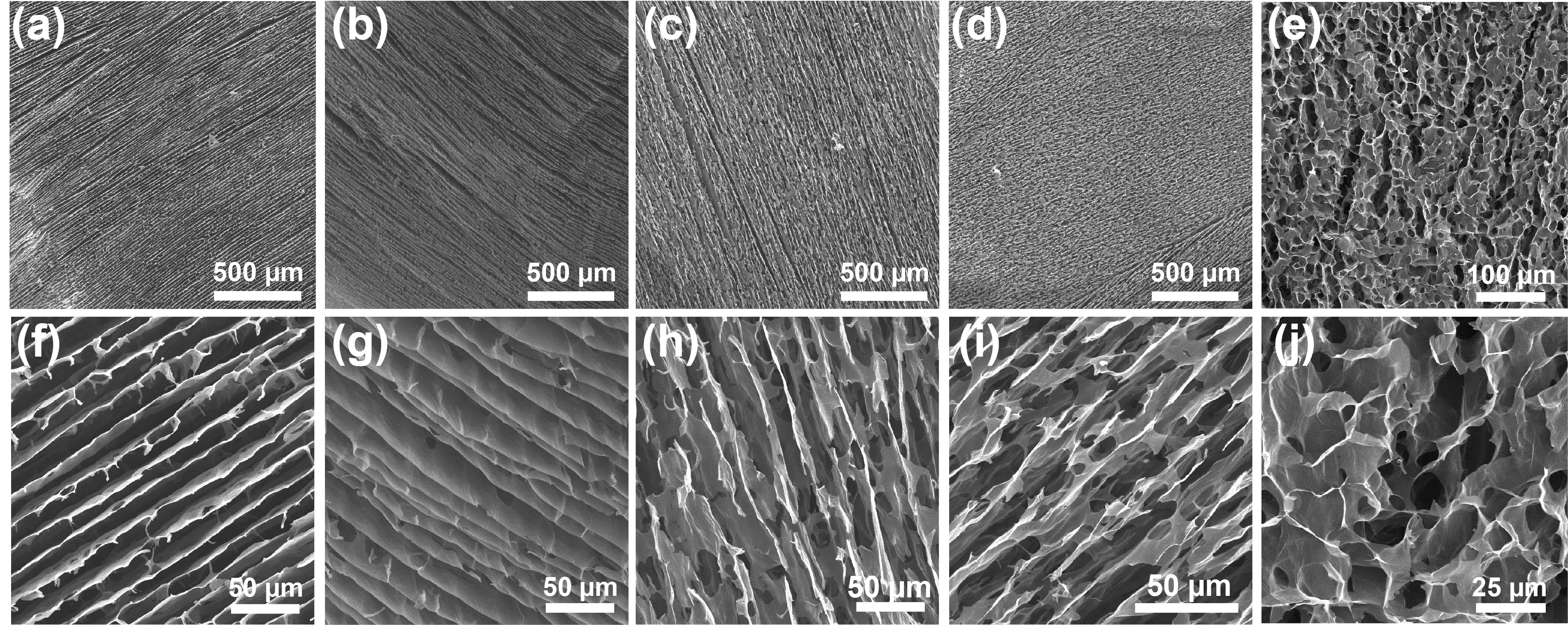


**Fig. S2**. (**a-e**) Low- and (**f-j**) high-magnification SEM images of morphologies of (**a, f**) P6G4, (**b, g**) P5G5, (**c, h**) P4G6, (**d, i**) P3G7, and (**e, j**) GO observed along Z-axis


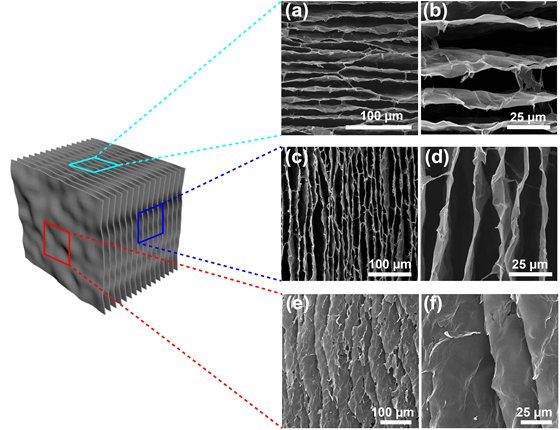


**Fig. S3.** SEM images of morphologies of P6G4-2800 observed along (**a, b**) Z-axis, (**c, d**) X axis, and (e, f) Y axis

**
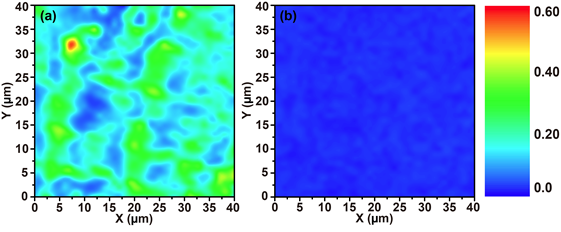
**

**Fig. S4.** Raman mapping images of (**a**) PAA-2800, and (**b**) GO-2800


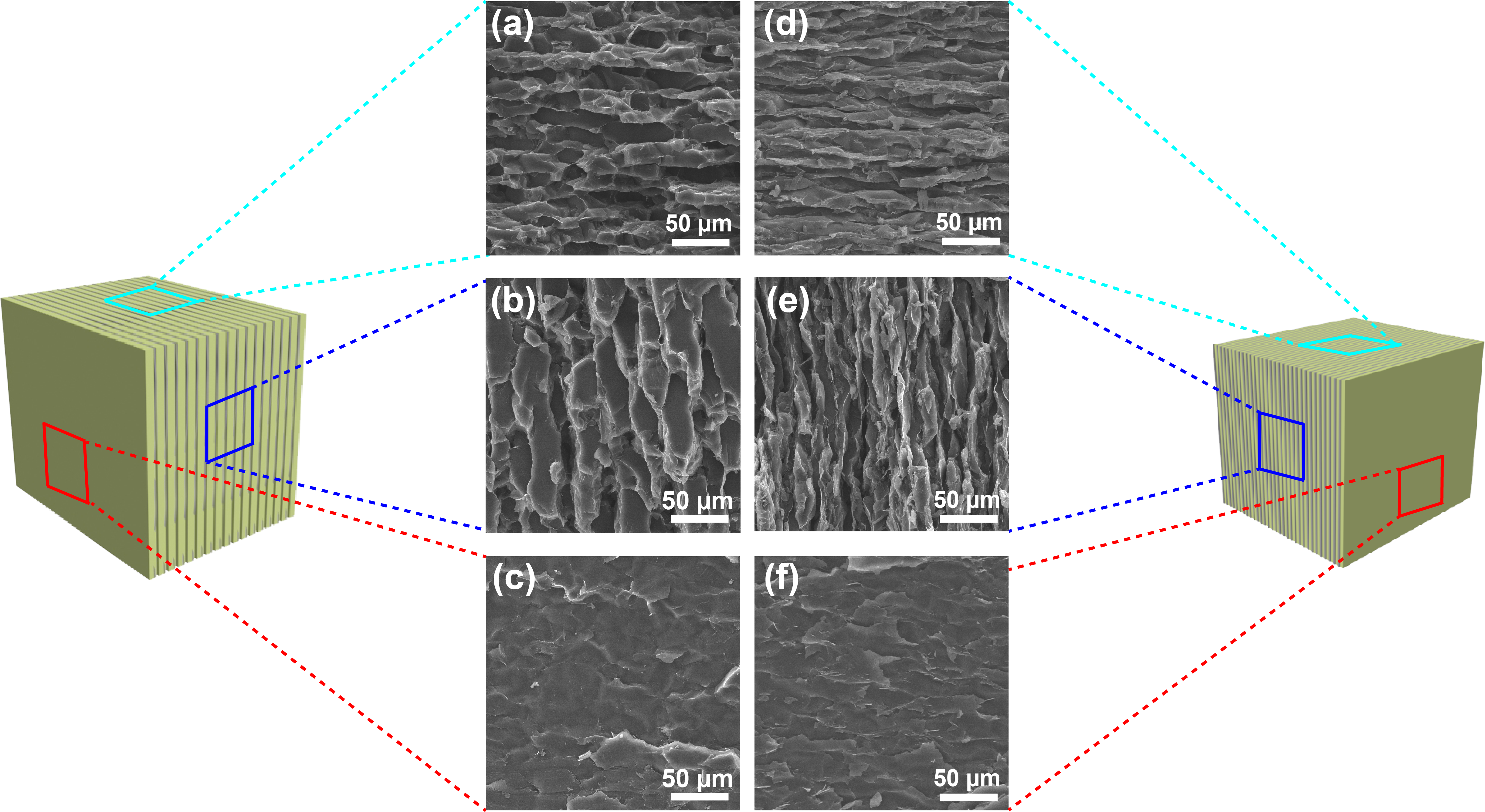


**Fig. S5.** SEM images of morphologies of (**a-c**) GE4 and (**d-f**) GE4-70% observed from three directions


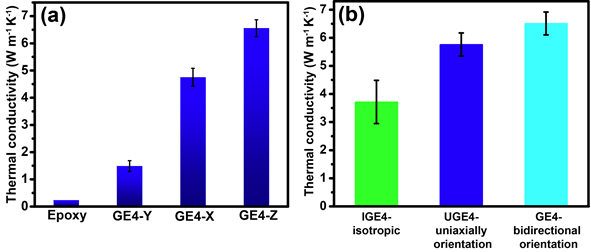


**Fig. S6.** (**a**) Comparison of thermal conductivities of GE4 in three directions. (**b**) Comparison of thermal conductivities of composites with isotropic aerogel (IGE4), and unidirectionally and bidirectionally orientated aerogels


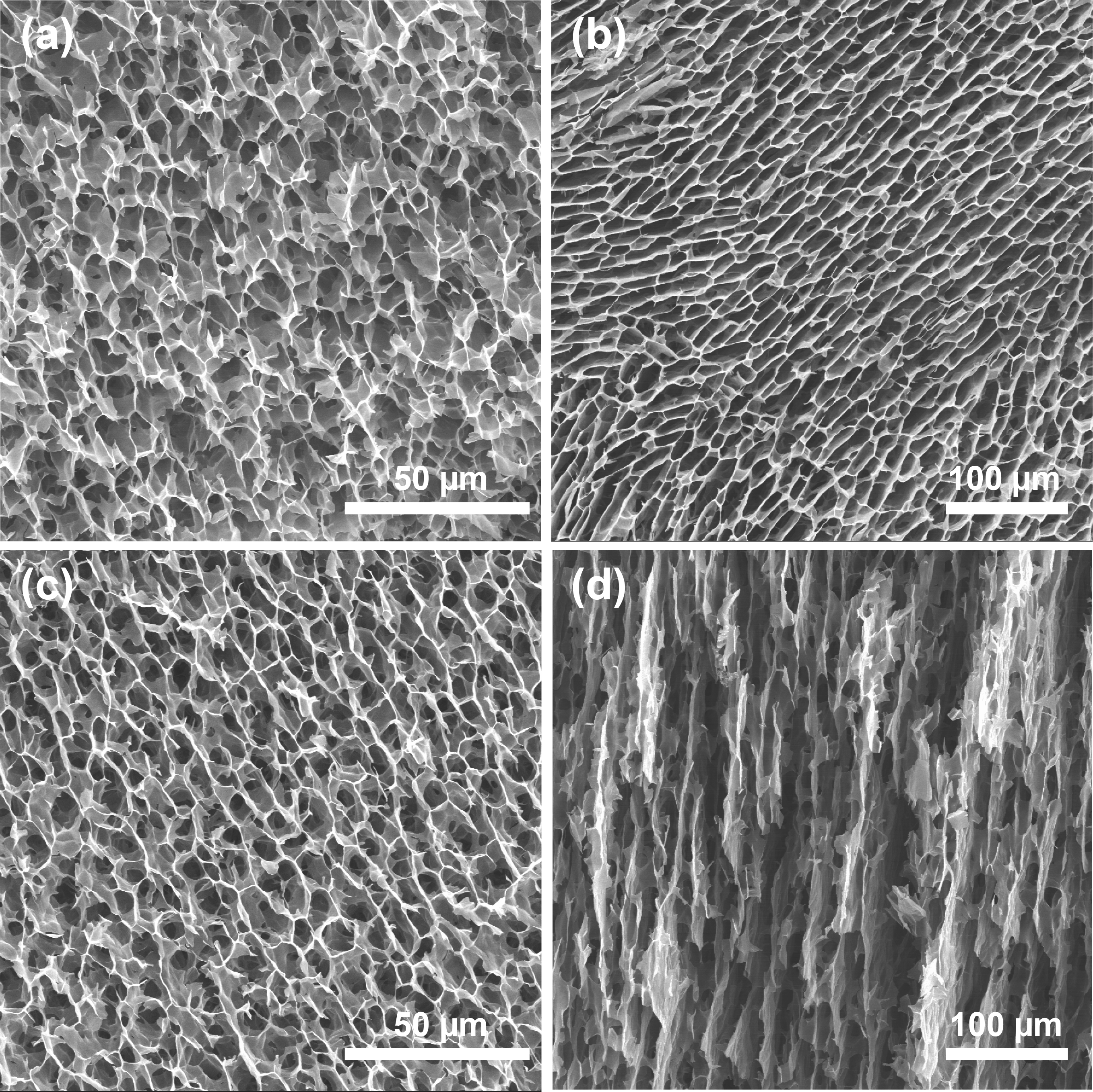


**Fig. S7.** Longitudinal view SEM images of (**a**) IP6G4-2800 and (**b**) UP6G4-2800. Transversal view SEM images of (**c**) IP6G4-2800 and (**d**) UP6G4-2800


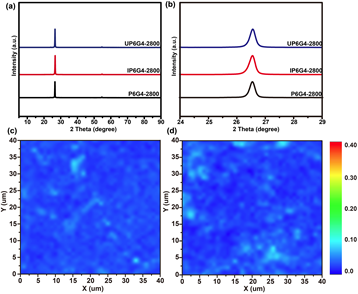


**Fig. S8.** (**a, b**) XRD patterns of P6G4-2800, IP6G4-2800 and UP6G4-2800. Raman mapping of (**c**) IP6G4-2800 and (**d**) UP6G4-2800. The average I_D_/I_G_ of IP6G4-2800 and UP6G4-2800 are ~0.030 and ~0.031, respectively


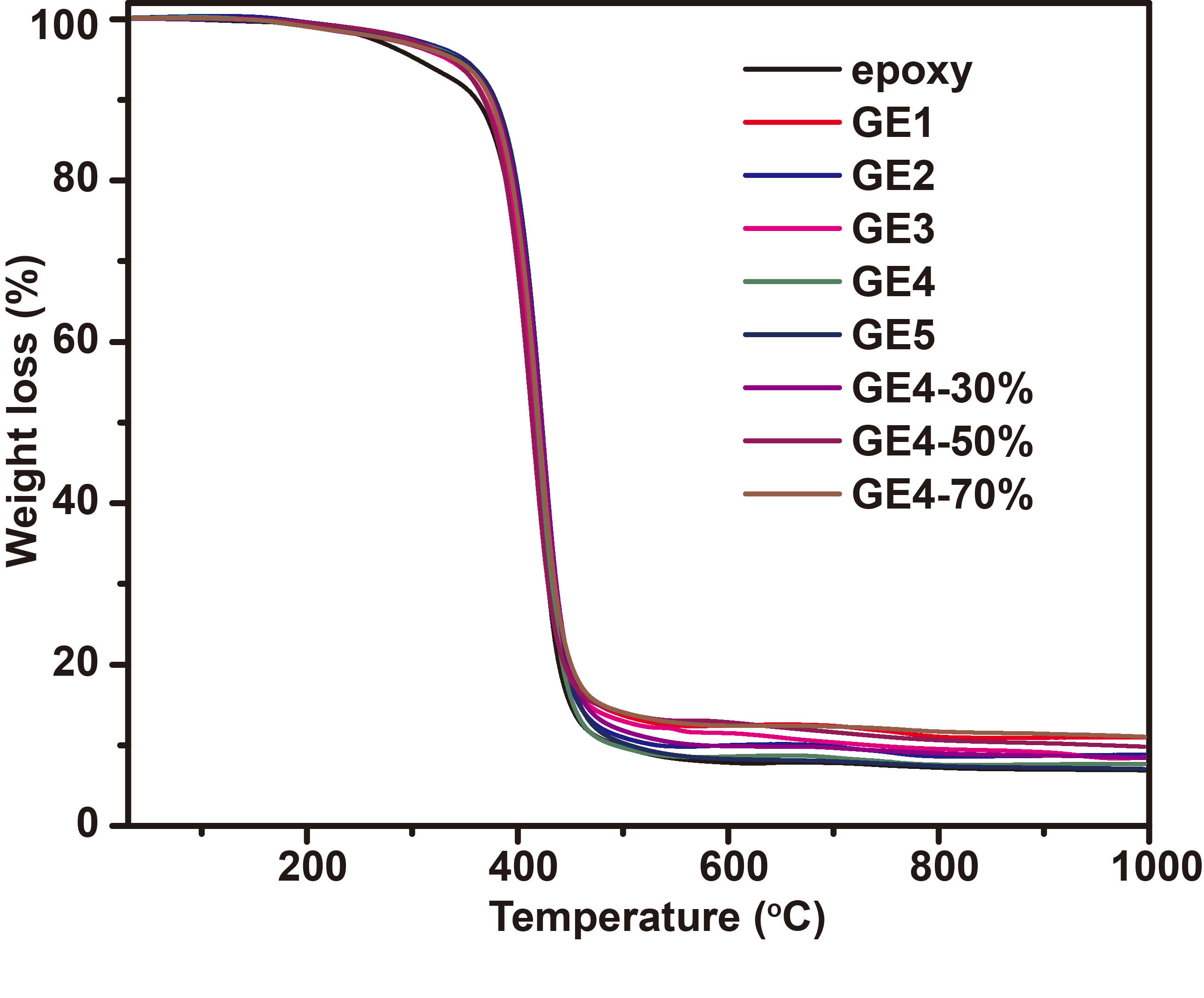


**Fig. S9.** TGA curves of epoxy and epoxy/LSGA composites


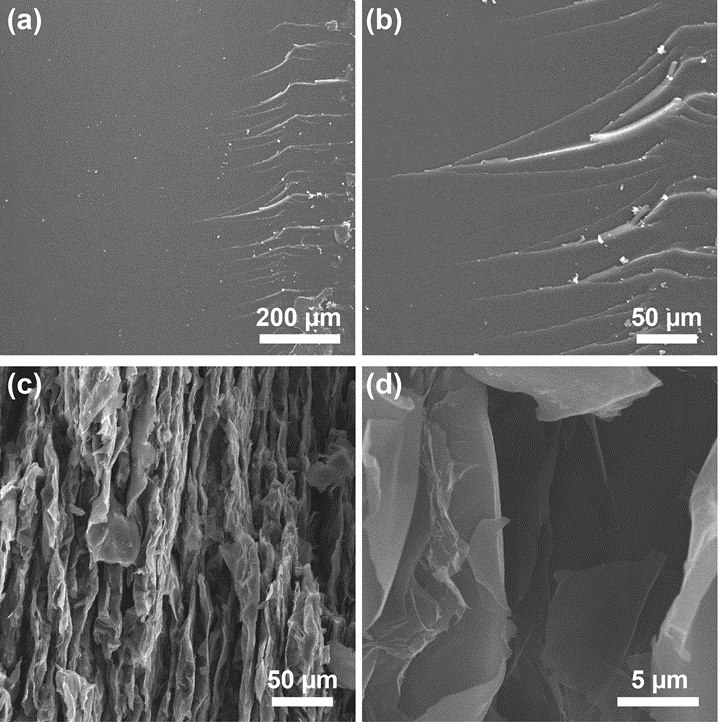


**Fig. S10.** Fracture surfaces of (**a, b**) epoxy and (**c, d**) GE4-70%

**Table S1. Detailed ingredients of PAAS/GO hybrid aerogels**

| **Aerogels** | **PAA (g)** | **TEA (g)** | **GO (g)** | **Water (g)** |
| --- | --- | --- | --- | --- |
| PAA | 1.60 | 0.77 | 0 | 37.63 |
| P9G1 | 1.44 | 0.69 | 0.16 | 37.71 |
| P8G2 | 1.28 | 0.61 | 0.32 | 37.79 |
| P7G3 | 1.12 | 0.54 | 0.48 | 37.86 |
| P6G4 | 0.96 | 0.46 | 0.64 | 37.94 |
| P5G5 | 0.80 | 0.38 | 0.80 | 38.02 |
| P4G6 | 0.64 | 0.31 | 0.96 | 38.09 |
| P3G7 | 0.48 | 0.23 | 1.12 | 38.17 |
| GO | 0 | 0 | 1.60 | 38.40 |

**Table S2. Filler contents, through-plane thermal conductivities of graphene/epoxy composites, and average I_D_/I_G_ values of LSGAs**

| **Composites** | **Filler content**  **(wt%)** | **Filler content**  **(vol%)** | **Average I_D_/I_G_ of LSGAs** | **Thermal conductivity**  **in direction Z (W m^-1^ K^-1^)** |
| --- | --- | --- | --- | --- |
| GE1 | 4.42 | 2.39 | 0.087 | 6.20±0.20 |
| GE2 | 2.29 | 1.22 | 0.072 | 6.53±0.33 |
| GE3 | 1.60 | 0.86 | 0.044 | 6.07±0.38 |
| GE4 | 1.23 | 0.68 | 0.036 | 6.51±0.41 |
| GE5 | 0.99 | 0.53 | 0.028 | 5.57±0.30 |
| GE4-30% | 1.65 | 0.88 | 0.036 | 7.66±0.69 |
| GE4-50% | 2.7 | 1.45 | 0.036 | 12.77±0.90 |
| GE4-70% | 4.28 | 2.30 | 0.036 | 20.03±1.11 |

**Table S3.** Comparison of thermal conductivities and specific TCE of our composites with those reported in the literature

| **Fillers** | **Matrix** | **Content (vol%)** | **K_∥_= in-plane**  **K_⊥_= through-plane**  **(W m^-1^ K^-1^)** | **Specific TCE** | **Ref.** |
| --- | --- | --- | --- | --- | --- |
| RGO | Epoxy | ~0.53 | 1.4 | ~1132 | [1] |
| GNP | Epoxy | 2.80 | 1.5 | ~244 | [2] |
| GNP | Epoxy | ~5.57 | 1.53 | ~119 | [3] |
| GNP/CNT | Epoxy | ~6.22 | 1.75 | ~125 | [4] |
| 3D graphene aerogel | Epoxy | 0.92 | K_⊥_= 2.13 | ~1332 | [5] |
| 3D BNNS network | Epoxy | ~9.29 | K_⊥_= 2.85 | ~181 | [6] |
| Graphene | SBR | 15.0 | 2.92 | ~90 | [7] |
| GNP | Octadecanol | ~3.83 | 3.55 | ~395 | [8] |
| 3D graphene foam | Wax | 1.23 | 3.6 | ~1500 | [9] |
| Graphene woven fabrics | PI | ~7.79 | K_∥_=3.73, K_⊥_= 0.41 | ~182 | [10] |
| 3D BNNS network | Epoxy | 34.0 | K_⊥_= 4.42 | ~66 | [11] |
| 3D graphene aerogel | Octadecanol | ~1.58 | 4.28 | ~1065 | [12] |
| 3D BN-RGO network | Epoxy | 13.16 | K_⊥_= 5.05 | ~206 | [13] |
| Graphene | Epoxy | 10.0 | 5.1 | ~230 | [14] |
| RGO/GNP aerogel | Octadecanol | ~4.67 | 5.92 | ~541 | [15] |
| Aligned BN aerogel | Epoxy | 15.0 | K_⊥_= 6.07 | ~196 | [16] |
| GNP/CNT | Epoxy | 50.0 | 7.3 | ~71 | [17] |
| Aligned graphene aerogel | Wax | ~1.31 | K_∥_=2.68, K_⊥_= 8.87 | ~1858 | [18] |
| Graphene flakes | PVDF | 25.0 | K_∥_=10.19 | ~196 | [19] |
| Graphene foam/  Graphene sheets | NR | 6.20 | K_∥_=10.64, K_⊥_= 3.0 | ~1300 | [20] |
| GNP | Epoxy | 25.0 | 12.4 | ~282 | [21] |
| 3D graphene aerogel | PDMS | ~5.34 | K_∥_=28.77, K_⊥_= 1.62 | ~2974 | [22] |
| GNP | Epoxy | ~6.60 | K_∥_=33.54 | ~2526 | [23] |
| Aligned graphene aerogel | Epoxy | 19.0 | K_∥_=17.1, K_⊥_= 35.5 | ~884 | [24] |
| Worm-like expanded graphite | Wax | ~16.0 | K_∥_=40 | ~1243 | [25] |
| BNNS | Aramid nanofiber | ~21.5 | K_∥_=46.7, K_⊥_= 0.13 | ~266 | [26] |
| **Aligned graphene aerogel** | **Epoxy** | **2.30** | **K_⊥_= 20.0** | **4310** | **This work** |

**Supplementary References:**

[S1] O. Eksik, S.F. Bartolucci, T. Gupta, H. Fard, T. Borca-Tasciuc et al., A novel approach to enhance the thermal conductivity of epoxy nanocomposites using graphene core–shell additives. Carbon **101**, 239-244 (2016). https://doi:10.1016/j.carbon.2016.01.095

[S2] X. Shen, Z. Wang, Y. Wu, X. Liu, Y.B. He et al., Multilayer graphene enables higher efficiency in improving thermal conductivities of graphene/epoxy composites. Nano Lett. **16**(6), 3585-3593 (2016). https://doi:10.1021/acs.nanolett.6b00722

[S3] S.H. Song, K.H. Park, B.H. Kim, Y.W. Choi, G.H. Jun et al., Enhanced thermal conductivity of epoxy-graphene composites by using non-oxidized graphene flakes with non-covalent functionalization. Adv. Mater. **25**(5), 732-737 (2013). https://doi:10.1002/adma.201202736

[S4] A. Yu, P. Ramesh, X. Sun, E. Bekyarova, M.E. Itkis, R.C. Haddon, Enhanced thermal conductivity in a hybrid graphite nanoplatelet–carbon nanotube filler for epoxy composites. Adv. Mater. **20**(24), 4740-4744 (2008). https://doi:10.1002/adma.200800401

[S5] G. Lian, C.-C. Tuan, L. Li, S. Jiao, Q. Wang et al., Vertically aligned and interconnected graphene networks for high thermal conductivity of epoxy composites with ultralow loading. Chem. Mater. **28**(17), 6096-6104 (2016). https://doi:10.1021/acs.chemmater.6b01595

[S6] X. Zeng, Y. Yao, Z. Gong, F. Wang, R. Sun et al., Ice-templated assembly strategy to construct 3D boron nitride nanosheet networks in polymer composites for thermal conductivity improvement. Small **11**(46), 6205-6213 (2015). https://doi:10.1002/smll.201502173

[S7] Y. Li, F. Xu, Z. Lin, X. Sun, Q. Peng et al., Electrically and thermally conductive underwater acoustically absorptive graphene/rubber nanocomposites for multifunctional applications. Nanoscale **9**(38), 14476-14485 (2017). https://doi:10.1039/c7nr05189a

[S8] G. Xin, H. Sun, S.M. Scott, T. Yao, F. Lu, et al., Advanced phase change composite by thermally annealed defect-free graphene for thermal energy storage. ACS Appl. Mater. Interfaces **6**(17), 15262-15271 (2014). https://doi:10.1021/am503619a

[S9] H. Ji, D.P. Sellan, M.T. Pettes, X. Kong, J. Ji et al., Enhanced thermal conductivity of phase change materials with ultrathin-graphite foams for thermal energy storage. Energy Environ. Sci. **7**(3), 1185-1192 (2014). https://doi:10.1039/c3ee42573h

[S10] J. Gong, Z. Liu, J. Yu, D. Dai, W. Dai et al., Graphene woven fabric-reinforced polyimide films with enhanced and anisotropic thermal conductivity. Compos. A Appl. Sci. Manuf. **87**, 290-296 (2016). https://doi:10.1016/j.compositesa.2016.05.010

[S11] J. Hu, Y. Huang, Y. Yao, G. Pan, J. Sun et al., Polymer composite with improved thermal conductivity by constructing a hierarchically ordered three-dimensional interconnected network of BN. ACS Appl. Mater. Interfaces **9**(15), 13544-13553 (2017). https://doi:10.1021/acsami.7b02410

[S12] J. Yang, X. Li, S. Han, R. Yang, P. Min et al., High-quality graphene aerogels for thermally conductive phase change composites with excellent shape stability. J. Mater. Chem. A **6**(14), 5880-5886 (2018). https://doi:10.1039/c8ta00078f

[S13] Y. Yao, J. Sun, X. Zeng, R. Sun, J.B. Xu et al., Construction of 3D skeleton for polymer composites achieving a high thermal conductivity. Small **14**(13), 1704044 (2018). https://doi:10.1002/smll.201704044

[S14] K.M. Shahil, A.A. Balandin, Graphene-multilayer graphene nanocomposites as highly efficient thermal interface materials. Nano Lett. **12**(2), 861-867 (2012). https://doi:10.1021/nl203906r

[S15] J. Yang, X. Li, S. Han, Y. Zhang, P. Min et al., Air-dried, high-density graphene hybrid aerogels for phase change composites with exceptional thermal conductivity and shape stability. J. Mater. Chem. A **4**(46), 18067-18074 (2016). https://doi:10.1039/c6ta07869a

[S16] J. Han, G. Du, W. Gao, H. Bai, An anisotropically high thermal conductive boron nitride/epoxy composite based on nacre‐mimetic 3D network. Adv. Funct. Mater. **29**(13), 1900412 (2019). https://doi:10.1002/adfm.201900412

[S17] X. Huang, C. Zhi, P. Jiang, Toward effective synergetic effects from graphene nanoplatelets and carbon nanotubes on thermal conductivity of ultrahigh volume fraction nanocarbon epoxy composites. J. Phys. Chem. C **116**(44), 23812-23820 (2012). https://doi:10.1021/jp308556r

[S18] P. Min, J. Liu, X. Li, F. An, P. Liu et al., Thermally conductive phase change composites featuring anisotropic graphene aerogels for real-time and fast-charging solar-thermal energy conversion. Adv. Funct. Mater. **28**(51), 1805365 (2018). https://doi:10.1002/adfm.201805365

[S19] H. Jung, S. Yu, N.S. Bae, S.M. Cho, R.H. Kim et al., High through-plane thermal conduction of graphene nanoflake filled polymer composites melt-processed in an l-shape kinked tube. ACS Appl. Mater. Interfaces **7**(28), 15256-15262 (2015). https://doi:10.1021/acsami.5b02681

[S20] Z. Wu, C. Xu, C. Ma, Z. Liu, H.M. Cheng et al., Synergistic effect of aligned graphene nanosheets in graphene foam for high-performance thermally conductive composites. Adv. Mater. **31**(19), 1900199 (2019). https://doi:10.1002/adma.201900199

[S21] M. Shtein, R. Nadiv, M. Buzaglo, K. Kahil, O. Regev, Thermally conductive graphene-polymer composites: size, percolation, and synergy effects. Chem. Mater. **27**(6), 2100-2106 (2015). https://doi:10.1021/cm504550e

[S22] H. Fang, Y. Zhao, Y. Zhang, Y. Ren, S.L. Bai, Three-dimensional graphene foam-filled elastomer composites with high thermal and mechanical properties. ACS Appl. Mater. Interfaces **9**(31), 26447-26459 (2017). https://doi:10.1021/acsami.7b07650

[S23] Q. Li, Y. Guo, W. Li, S. Qiu, C. Zhu et al., Ultrahigh thermal conductivity of assembled aligned multilayer graphene/epoxy composite. Chem. Mater. **26**(15), 4459-4465 (2014). https://doi:10.1021/cm501473t

[S24] F. An, X. Li, P. Min, P. Liu, Z.G. Jiang et al., Vertically aligned high-quality graphene foams for anisotropically conductive polymer composites with ultrahigh through-plane thermal conductivities. ACS Appl. Mater. Interfaces **10**(20), 17383-17392 (2018). https://doi:10.1021/acsami.8b04230

[S25] S. Wu, T. Li, Z. Tong, J. Chao, T. Zhai et al., High-performance thermally conductive phase change Ccomposites by large-size oriented graphite sheets for scalable thermal energy harvesting. Adv. Mater. **31**(49), 1905099 (2019). https://doi:10.1002/adma.201905099

[S26] K. Wu, J. Wang, D. Liu, C. Lei, D. Liu et al., Highly thermoconductive, thermostable, and super-flexible film by engineering 1D rigid rod-like aramid nanofiber/2D boron nitride nanosheets. Adv. Mater. **32**(8) 1906939 (2020). https://doi:10.1002/adma.201906939
